# Review study of alteration functional activities and networks in ulcerative colitis using resting-state fMRI

**DOI:** 10.3389/fneur.2025.1608371

**Published:** 2025-08-19

**Authors:** Ali S. Alyami

**Affiliations:** Diagnostic Radiography Technology Department, College of Nursing and Health Sciences, Jazan University, Jazan, Saudi Arabia

**Keywords:** resting-state fMRI, ulcerative colitis, resting-state networks, functional connectivity, brain-gut axis

## Abstract

**Background:**

Ulcerative colitis (UC), a chronic inflammatory bowel disease (IBD), is linked to neuropsychiatric comorbidities and changes in brain connectivity through the brain-gut axis. Resting-state functional MRI (RS-fMRI) offers a non-invasive approach to examining these neural alterations; however, no comprehensive review has compiled findings specific to UC.

**Objective:**

This review summarizes RS-fMRI studies to characterize functional connectivity (FC) alterations and methodological approaches in UC patients compared to healthy controls (HCs) and other inflammatory bowel disease (IBD) subtypes.

**Methods:**

Literature searches were performed in Ovid, PubMed, Google Scholar, and EMBASE (up to July 2025) using keywords: resting-state fMRI, RS-FMRI, ulcerative colitis, and UC. Few studies meeting the inclusion criteria (human participants, UC diagnosis, RS-fMRI analysis) were reviewed.

**Key findings:**

Seven studies were included in this review. UC patients show disrupted FC in the salience, cerebellar, visual, default mode, and dorsal attention networks. Reduced hippocampal activity is linked to working memory deficits, while increased FC in corticolimbic areas (e.g., caudate anterior, cingulate) correlates with active inflammation. Grey matter volume decreases in cerebellar regions and increases in parahippocampal regions. Sex-specific differences in FC are observed, especially in the visual and attention networks. Altered FC patterns are associated with the severity of anxiety, depression, and stress. UC exhibits distinct neural signatures compared to CD.

**Implications:**

RS-fMRI uncovers UC-specific neural phenotypes, advancing the mechanistic understanding of brain-gut interactions. These findings highlight potential biomarkers for neuropsychiatric comorbidities and support the use of integrated fMRI in clinical assessments. Future research should focus on longitudinal studies, larger cohorts, and AI-enhanced analytics to clarify causality and identify therapeutic targets.

## 1 Introduction

Inflammatory Bowel Disease (IBD) includes two primary chronic, immune-mediated inflammatory disorders: Crohn’s disease (CD) and ulcerative colitis (UC). These disorders are characterized by persistent inflammation of the gastrointestinal tract, though they differ in their pathological features and anatomical distribution (1, 2). The exact cause of IBD is not fully understood. However, it is believed to result from a combination of genetic predisposition, environmental factors, an abnormal immune response and changes in the gut microbiome (3–5). IBD can affect individuals of any age, but it is most commonly diagnosed in teenagers and young adults (6). The condition often presents with signs such as weight loss, persistent diarrhea, abdominal pain, rectal bleeding and fatigue (7–9).

The global prevalence of UC—a second type of IBD that primarily affects the colon—is on the rise, with the highest incidence rates found in the United States, Canada, Northern Europe and Australia (10, 11). UC can occur at any age, from early childhood to older adulthood, and it is characterized by alternating periods of remission and exacerbation (12). A thorough understanding of the underlying pathophysiology of UC remains elusive despite ongoing research. However, researchers have proposed several factors that may contribute to the development and progression of UC, including abnormal immune responses, microbiomes, and environmental and genetic factor (13, 14).

The term “brain-gut axis” refers to the intricate interactions among neuroendocrine pathways, the peripheral, central and autonomic nervous systems, as well as the gastrointestinal system (15). The bidirectional communication between these factors plays a critical role in modulating IBD pathophysiology and clinical outcomes (15, 16). An in-depth exploration of the intricate mechanisms that support the brain-gut axis is beyond the scope of this review. In brief, emerging evidence highlights that psychological stress, affective disorders (e.g., depression, anxiety), and altered neuroimmune signaling pathways may exacerbate intestinal inflammation, impair mucosal healing, and amplify symptom burden, thereby contributing to disease progression and severity. It has been established for some time that psychological stress can exacerbate disease activity in IBD. Recent research suggests that stress-induced modifications in gastrointestinal inflammation may be influenced by changes in bacterial-mucosal interactions, dysregulation of the hypothalamic–pituitary–adrenal axis, mucosal mast cell activity, and mediators such as corticotropin-releasing factor (16). Patients suffering from UC frequently exhibit mental symptoms, including depression and anxiety, which may stem from the disease’s chronicity and interference with daily activities (17, 18). Recently, the correlation between inflammatory activity and psychological morbidity has aroused interest. A recent meta-analysis (19), reported the prevalence of anxiety and depression in people with UC at 32.6 and 23%, respectively. In patients with active UC, 70.8% had anxiety and 41.3% had depression (19). People with UC exhibit a higher prevalence of mood disturbances, alterations in affect and psychiatric disorders than the general population (20, 21). Evidence indicates that the stress response and psychological stressors may contribute to disease activity through the brain-gut axis; however, there has been no focused research on the impact of depression or anxiety on inflammation.

Functional magnetic resonance imaging (fMRI) is an imaging technique that measures changes in oxygenation and blood flow in various brain regions. Recent advancements in blood oxygenation level-dependent (BOLD) functional magnetic resonance imaging (BOLD-fMRI) have facilitated the non-invasive identification of brain activity alterations, including altered spontaneous neural activity in specific brain regions and its association with physiological states. There are two main categories of inspection techniques that fMRI encompasses: task-based state and resting-state. Task-state fMRI (T-fMRI) has been the predominant approach for localizing language and motor functions by examining alterations in the BOLD signal during the execution of specific tasks or activities (22). Resting-state fMRI (RS-fMRI) utilizes inherent low-frequency fluctuations, typically below 0.1 Hz, to detect alterations in BOLD signals that occur during periods of mental activity or minimal physical activity, without implementing task-specific designs or stimuli (23).

In recent times, functional and structural brain MRI technology has become widespread in computer-aided IBD diagnoses. An increasing body of evidence from multiple studies has demonstrated that disturbances in the brain–gut axis and central nervous system (CNS) dysfunction play significant roles in the underlying mechanisms of IBD (24, 25). Functionally, people with IBD showed notable BOLD signal reductions in the cerebellum, thalamus and amygdala when compared to the HC group (26). Several studies have explored brain structure and function in CD are increasingly being reported using fMRI (27–30). In addition, a few studies using RS-fMRI to distinguish UC from CD are still in their early stages, and there is limited literature directly comparing the two conditions. However, some preliminary studies suggest that differences in FC patterns may exist, particularly in brain regions associated with pain processing, emotional regulation, and visceral sensitivity. Inconsistent findings were reported when comparing UC to CD. For example, Kornelsen et al. (31), found that no significant differences in grey matter volume were observed in the medial frontal cortex when comparing CD to UC. However, they found that the CD group exhibited a significant increase in grey matter volume in the inferior frontal area and the left cerebellum compared to the UC group. An FC between the dorsal attention and visual areas was found in both IBD patients and HC, as well as in the comparisons between UC and CD. Notably, the strength of this connectivity was approximately twice as pronounced in the UC group compared to the CD group. However, few studies have explored altered brain structures and functions in UC using fMRI (26, 32, 33). In this study, I primarily review existing research on RS-fMRI in UC, analyzing the changes in brain connectivity and functionality between patients with UC and HC participants. All relevant papers on this topic in English from the Ovid, PubMed, Google Scholar and EMBASE databases were reviewed.

## 2 The significance of studying FC patterns in UC

RS-fMRI captures intrinsic brain activity during periods of rest, independent of task performance. This offers a less onerous approach for patients, facilitates the investigation of interregional communication in the brain, and provides insights into underlying FC patterns. A resting-state network is a collection of brain regions that are anatomically separated but exhibit FC and engage in ongoing communication (34). By comparing FC patterns among patients with UC during remission stages and active disease, as well as among HCs, potential neural signatures or biomarkers can be identified. Analysing changes in connectivity patterns in response to therapeutic interventions can also contribute to the development of personalized treatment approaches.

## 3 Resting-state methods in IBD

RS-fMRI has emerged as a valuable tool in studying the neurobiological impacts of IBD. The application of RS-fMRI in IBD research involves several unique considerations compared to traditional brain cognition and brain disease studies. These differences span experimental setups, acquisition parameters, and analysis methods, highlighting the unique challenges and goals of studying a condition characterized by gut-brain interactions. For example, while both IBD and traditional studies use similar MRI field strengths, IBD research may focus on specific pulse sequences, such as echo planar imaging (EPI), to achieve shorter acquisition time (allowing better temporal resolution). Moreover, it is attractive due to both its BOLD contrast sensitivity and imaging speed, but is also associated with diminished image quality and inherent artifacts (35).

In terms of experimental design, there are several variables associated with each study (such as demand for data handling, availability of imaging instruments, the specific nature of the research question and cost) that make it essential to ensure statistical efficiency of the analysis and optimize BOLD signal acquisition time. Compared to conventional MRI, there is no one optimal design that will cover all RS-fMRI studies. Acquisition protocols in IBD research may include additional physiological monitoring to account for gastrointestinal-related factors like bowel activity, which can affect brain connectivity during scanning. One of the most crucial factors in optimizing an fMRI protocol regarding timing is the interval between the excitation of the slices and the subsequent signal acquisition, commonly referred to as echo time (TE). The selection of TE to enhance BOLD contrast is influenced by tissue properties and the strength of the magnetic field. Ideally, the chosen TE should correspond to the apparent T2* of the tissue being examined. For a magnetic field strength of 3 T, the TE is generally set to approximately 30 ms, with a typical range between 25 and 40 ms. (36, 37)

**Figure 1:**
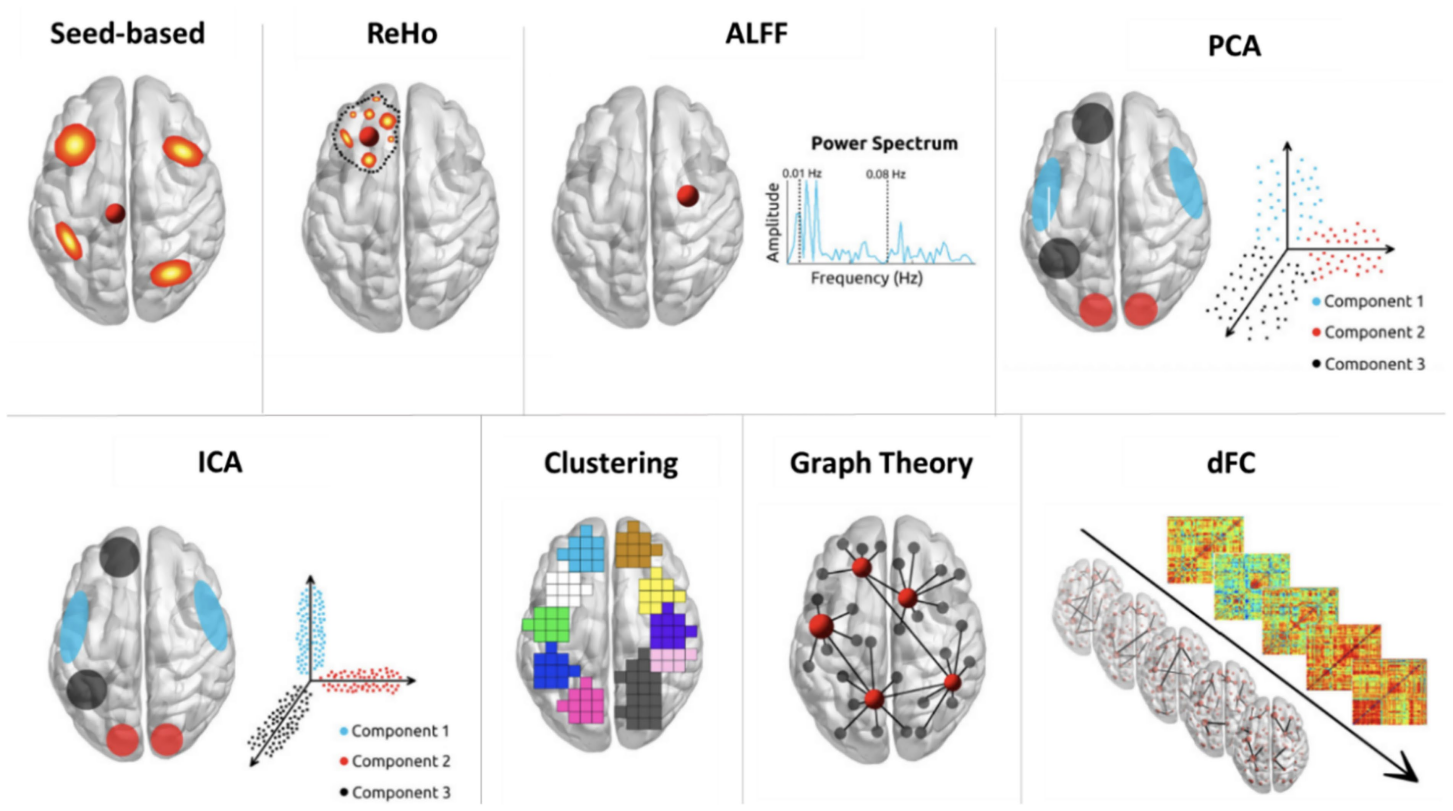
RS-fMRI methods. The top row (from left to right) illustrates various analytical approaches: Seed-Based Correlations, Regional Homogeneity (ReHo), Amplitude of Low-Frequency Fluctuations(ALFF), and Principal Component Analysis (PCA). The bottom row (from left to right) presents additional methods: Independent Component Analysis (ICA), Clustering, Graph Theory, and Dynamic Functional Connectivity (dFC). This figure is adapted from Soares et al. (39).

There are currently several methods for performing RS-fMRI analyses in IBD, such as FC, amplitude of low-frequency fluctuation (ALFF), regional homogeneity (ReHo), independent component analysis (ICA), graph theory, seed-based analysis principal component analysis (PCA) and region of interest (ROI)-to-ROI. FC is a quantitative measure that captures the degree of correlation between the time series of two distinct brain areas using linear temporal correlation methods. The ALFF method identifies voxel-level differences in the BOLD signal’s overall power, specifically targeting extremely low frequencies. ReHo uses Kendall’s coefficient of concordance to quantify the coherence or similarity between the time series of a given voxel and its neighbouring voxels. Graph theoretical analysis provides a computational framework for quantifying the topological architecture of large-scale brain networks, characterizing system-level properties such as segregation (modularity), integration (global efficiency), and small-world organization to explain the neurobiological principles underlying network optimization and information transfer efficiency (38). Figure 1 illustrates commonly used analysis methods in RS-MRI studies (39). As summarized in Table 1, each of these data processing techniques presents distinct advantages and disadvantages (40, 41).

**Table 1:** Demonstrates the advantages and disadvantages of some of the RS-fMRI analysis methods (41).

Methods	Advantage	Disadvantage
Amplitude of Low-Frequency Fluctuations	Can serve as a potential factor when examining FC and network dynamics. Characterized by its simplicity, it lacks any underlying hypotheses.	The fractional amplitude of the low-frequency fluctuations method is a preferable option due to its sensitivity to physiological noise (41).
Seed-Based FC Analysis	Has computational simplicity and the results can be interpreted intuitively.	Its reliance on seed selection renders it susceptible to bias (41).
Independent Component Analysis	Requires few a priori assumptions.	Presents brain networks one by one. Differentiating between physiological signals and noise can be challenging. The components extracted by ICA may not have clear biological interpretations (41).
Graph Analysis	This process can be conducted automatically using existing software, without any *a priori* assumptions and with minimal bias. Network-level insights: Captures a local (e.g., node centrality) and global (e.g., efficiency, modularity) properties of brain networks (59). Provides a robust framework for characterizing the topological organization of large-scale structural and functional brain networks, enabling the quantification of system-level properties such as the identification of highly interconnected hub regions critical for global information integration, modular community structure and small-world architecture (38)	Interpretation can be challenging (41).

## 4 Materials and methods

### 4.1 Search strategy

To achieve the objective of this comprehensive review, this review analyzed the most representative human studies from the past few decades that employed RS-fMRI in UC. An exhaustive search of the available English-language publications related to this topic was performed using the Ovid, PubMed, Google Scholar, and EMBASE databases. The following keywords were employed: functional MRI or resting-state fMRI and ulcerative colitis or UC covering the entire span of these databases up to July 2025.

### 4.2 Eligibility criteria

Articles published in English, involving participants diagnosed with UC, including UC or IBD patients and HCs, were included. However, studies conducted on animals, unpublished abstracts, comments, meeting summaries, guidelines, and reviews were excluded from this review. Furthermore, studies that did not specify the subtype of IBD were also excluded from consideration (Table 2). The reference lists of the retrieved articles were manually screened to identify any additional studies that met the predefined eligibility criteria.

**Table 2:** Study classification by cohort type.

Reference	Cohort type	UC-specific?	Mixed IBD?
(33)	UC vs. HC	Yes	No
(47)	UC vs. HC	Yes	No
(46)	UC vs. IBS vs. HC	Yes	No
(32)	UC vs. HC	Yes	No
(31)	UC + CD vs. HC	No	Yes (stratified)
(45)	UC + CD vs. HC	No	Yes (stratified)
(48)	UC + CD vs. HC	No	Yes (stratified)

## 5 Functional connectivity alterations reported in UC

Alterations in multiple resting-state networks—namely the cerebellar network (CN), visual network (VISN), sensory-motor network (SMN), default mode network (DMN), frontal–parietal network (FPN), dorsal attention network (DAN), salience network (SN) and executive control network (ECN)—have been implicated in UC (31). Moreover, RS-fMRI studies have revealed alterations in FC within and between key brain networks, such as the DMN and the SN, in IBD patients with depression (42). The few existing studies investigating altered FC in UC compared to HC have yielded inconsistent findings and a lack of consensus on these methods (43). Agostini et al. reported that they could not detect grey matter volume differences in patients with UC compared to HC (43). However, people with UC exhibited greater cortical thickness in specific subregions of the primary somatosensory cortex and anterior cingulate than patients with IBS and HCs (44). In terms of brain function, UC patients exhibited reduced thalamus and amygdala activity when exposed to positive emotional visual stimuli (26).

Wang et al. (33) employed RS-fMRI to characterize alterations in spontaneous neural activity among individuals with mild-to-moderate UC relative to HC cohorts. They also conducted seed-to-voxel FC mapping to characterize aberrant neural network interactions in patients stratified by ALFF-defined regional neural activity profiles (33). The study results indicated significant deviations in the limbic system, specifically in the bilateral hippocampus/parahippocampus and left posterior cingulate cortex. The ALFF measures were higher in the left middle frontal gyri and left posterior cingulate cortex but lower in the bilateral parahippocampal region. With hippocampal/parahippocampal as the seed point, the strengths of the FC in the left caudate nucleus, anterior cingulate cortex and the bilateral middle frontal gyri increased; using the left posterior cingulate cortex as the seed point, the strengths of the FC in the left angular gyrus and the middle cingulate cortex increased. Patients with UC who have active inflammation have shown increased FC in corticolimbic regions involving the left caudate nucleus, anterior cingulate cortex and bilateral middle frontal gyrus. However, patients with UC also showed reduced activity in the hippocampus/parahippocampus and prolonged reaction times in the two-back test. These findings suggest that working memory impairments in UC patients may be correlated with reduced hippocampal/parahippocampal activity. Moreover, relative to HC participants, individuals with UC exhibited statistically significant reductions in health-related quality of life, as evidenced by markedly lower scores on the Inflammatory Bowel Disease Questionnaire. They also found that the Perceived Stress Scale, Visual Analog Scale and Pittsburgh Sleep Quality Index scores of the HC group were significantly lower than those of the UC group. Assessment of standardized psychological measures and neurocognitive evaluations identified significant impairments in both cognitive and emotional domains among individuals with UC relative to HC.

Kornelsen et al.’s [32]prospective study investigated brain structure and function alterations in 76 participants with UC and 74 HCs using RS-fMRI. They reported significant brain structure and function differences between patients with UC and HCs. UC patients exhibited structural abnormalities in specific regions compared to HCs. Structural changes were predominantly observed in cognitive and emotional processing areas, such as the cerebellum and parahippocampal gyri. The UC group exhibited significantly higher grey matter volumes in both bilateral parahippocampal regions than the HC group. This difference remained significant in the right parahippocampus, even after applying the family-wise error correction. This study also found changes in the FC patterns of patients with UC. The study found reduced FC in the cerebellum and between the cerebellum and other brain networks and regions. In the cerebellum, the analysis of the ROI revealed a weaker FC between the anterior and posterior cerebellar nodes in individuals with UC than in HCs. The ICA demonstrated decreased FC in the CN of UC participants when compared to HCs. The FC between the VISN and bilateral parahippocampal regions demonstrated significant differences among members of the UC group. Specifically, decreased FC was observed in females with UC than in males. There are also clear differences in the limbic, cerebellar and visual cortex FC that are associated with UC. A multimodal brain MRI analysis revealed a strong association between behavioural symptoms and structural and functional alterations in deep grey matter, which influences stress and cognitive and emotional responses in IBD patients. Compared to HCs, patients with UC or CD demonstrated increased hypothalamus and amygdala volumes, neurodegeneration in the pallidum and putamen, and significantly increased activity and FC in brain regions associated with emotional and cognitive processing, including the limbic system, basal ganglia and hypothalamus (45).

Another recent study examined changes in functional brain connectivity and cortical stability in the brains of patients with UC compared to a HC and a group of irritable bowel syndrome (IBS) patients using RS-fMRI (46). The study identified potential correlations between these alterations and the relevant clinical parameters associated with UC. Functional brain rewiring pertains to the brain’s global plasticity or reconfiguration in response to a disease condition. The study included 74 participants in each group. The researchers found that the UC group showed higher FC than the IBS and HC groups. The network modularity analysis revealed significant differences among the three groups (*F* = 5.846, *p* = 0.003), and the posthoc analysis demonstrated that the UC group exhibited notably lower modularity than the HC (*p* = 0.015; Cohen’s d = 0.445) and IBS (*p* = 0.007; Cohen’s d = 0.480) cohorts. The dynamic functional connectome analysis revealed a significant correlation between the increased stability of the left medial prefrontal cortex in the UC group and high levels of perceived depression, stress and trait anxiety. In the UC group, no statistically significant associations were found between disease duration and mean modularity Q, global efficiency and FC at the global level. Similarly, no significant correlations were observed between disease duration and the nine nodal eigenvector centrality values at the nodal level.

Ren et al. (47) conducted an ICA-based resting-state fMRI study to characterize FC alterations in UC. Their study included 22 UC patients (9 in active phase, 13 in remission) and 23 matched HCs. Mood evaluations involved the Hamilton Depression Rating Scale (HAMD) and the Hamilton Anxiety Scale (HAMA). They found that increased intranetwork FC within the auditory network (AN), particularly in the left Rolandic operculum, and left superior temporal gyrus, orbitofrontal cortex and right temporal pole. Notably, heightened connectivity in the left Rolandic operculum persisted only in remission-phase patients, whereas no significant intranetwork differences were found during active disease. For internetwork connectivity, UC patients showed increased coupling between the anterior and posterior DMNs and improved connectivity between the left frontoparietal network and the dorsal attention network. Subgroup analyses revealed that UC patients in remission phases had further increased internetwork FC between the anterior and posterior DMNs but decreased connectivity between the left and right frontoparietal networks. In contrast, active-phase patients exhibited significantly increased internetwork FC between the dorsal attention network and the left frontoparietal network, along with decreased FC between the anterior DMN and the left frontoparietal network. Surprisingly, no significant correlations were found between disease severity (Mayo scores) or psychological symptoms (HAMA/HAMD scores) and FC changes within or between networks during different stages.

In another study, the same researchers conducted a comparative analysis of functional and structural brain images between patients with IBD (*n* = 11) and HCs (*n* = 74). Additionally, within the IBD cohort, they differentiated between 76 participants diagnosed with UC and 35 participants diagnosed with CD (31). The study found that there was a significant difference in FC in the components with spatial matches to the DAN, SMN, VISN, DMN, SN and CN for the IBD vs. HC contrast. The results further demonstrated increased thalamic grey matter volume alongside reduced FC between the middle temporal cortex and medial frontal regions, particularly involving the temporal pole and orbitofrontal cortex (cluster 1), as well as the inferior temporal gyri (cluster 4). The findings also identified diminished FC between the medial frontal cortex and the temporal fusiform gyri (cluster 7), as well as between the parahippocampal region and the posterior cingulate with the temporal pole and orbitofrontal cortex (cluster 5). The authors also reported that no statistically significant differences were detected regarding the relationship between FC and disease duration or Hospital Anxiety and Depression Scale scores between patients with CD and UC. In the component exhibiting spatial similarity to the DMN, a notable decrease in FC was observed in the left cerebellum in the IBD group compared to the HC group. The IBD group also exhibited reduced FC between a cluster situated in the left lateral occipital cortex and the VIS component while displaying a significant increase in FC in another VIS component involving the right lateral occipital cortex. Notable variations in FC were identified in the two components related to the DAN when comparing the HC and IBD groups. Specifically, altered FC was reported with the right angular gyrus in one component, whereas another network exhibited connectivity changes involving the right lateral occipital region and the occipital pole.

A recent study has observed differences between IBD (*n* = 46; *n* = 15 with UC and *n* = 31 with CD) in stable remission and HC (*n* = 17), showing functional alterations in the inferior temporal, inferior as well as medial frontal, subcallosal gyrus and rectal (ALFF) and the superior frontal gyrus (ReHo), and structural changes in middle frontal and temporal regions (VBM) (48). They also found that the depression scores were marginally elevated in the patient group, with the highest scores observed in those with CD. However, the mean scores remained below the threshold indicative of clinically relevant depressive symptoms. Additionally, no statistically meaningful variations in fatigue or anxiety manifestations were observed when comparing individuals with IBD to HCs. Table 3 presents some studies that used RS-fMRI in UC.

**Table 3:** Summaries of some studies using RS-fMRI in UC.

Ref.	Participants (n)	Disease activity	Analysis methods	Key findings
(33)	UC: 41 HC: 42	Active (Mayo Score)	ALFF Seed-to-voxel FC	↑FC in corticolimbic regions (left caudate, anterior cingulate cortex, bilateral middle frontal gyri) in patient with UC. ↓ALFF in bilateral hippocampus/parahippocampus in UC patient. ↑ ALFF in the left posterior cingulate cortex and the left middle frontal gyrus. In patient with UC working memory deficits linked to hippocampal activity ↓
(32)	UC: 76 HC: 74	Mixed (Active/Remission)	ICA ROI-to-ROI VBM	↑Gray matter in bilateral parahippocampi in UC compared to HC. Significant differences in FC were observed between groups in multiple networks, including the DAN, VISN, DMN and CN. Sex differences in DAN/VISN connectivity
(47)	UC: 22 HC: 23	Mixed (Active/Remission)	ICA	↑Internetwork FC between the anterior and posterior DMNs in UC ↑Internetwork FC between the left frontoparietal network and the dorsal attention network in UC In UC patients, no significant correlations were found between psychological scales and altered brain regions
(46)	UC: 74 IBS: 74 HC: 74	Mixed (Active/Remission)	Graph theory Dynamic FC	↑Global FC, ↓modularity in UC ↓Eigenvector centrality in ECN regions (HC > IBS > UC) ↓ DMN-ECNs connectivity, salience/ventral and dorsal attentions in UC compared to HC. ↑ Connections between the somatomotor subnetwork and dorsal attention in UC compared to HC. ↑ increased middle-range connections and reduced short-range connections compared to the HC and IBS groups.
(31)	UC: 76 CD: 35 HC: 74	Mixed (Active/Remission)	• ICA • VBM • ROI-to-ROI	↓FC in DMN (left cerebellum) Significant differences identified by all three analysis methods in the default mode, CN and VISN in all groups. ↓FC in the left cerebellar clusters and ROI clusters, largely involving the bilateral cerebellar V, VI and Crus I in IBD compared to HC. Structural/functional differences between UC/CD
(45)	UC: 9 CD: 26 HC: 32	Mixed (Active/Remission)	• ALFF • ROI-to-ROI	↑Activity and connectivity in emotional processing regions including parts of the hypothalamus basal ganglia and limbic system in IBD comapred to HC ↑Hippocampal ALFF in active disease Sex-specific FC reductions (hippocampus-thalamus) in Females.
(48)	UC: 15 CD: 31 HC: 17	Inactive (Remission)	ReHo ALFF ICA VBM	Frontotemporal alterations in IBD (most in UC) No symptom correlations Multimodal fusion changes

## 6 Synthesis of consistent patterns and key contradictions

The literature review reveals consistent patterns in brain structure and function in IBD patients. Changes in the DMN and disruptions in cerebellar function are common findings across multiple studies. Kornelsen et al. (31) reported structural and functional brain changes associated with IBD, highlighting the importance of the DMN. Similarly, Kornelsen et al. (32) and Ren et al. (47) found alterations in the DMN in UC patients, suggesting a possible link between neural connectivity and gut inflammation within this brain network. Fan et al. [33]confirmed these results by demonstrating abnormal brain activity in active-stage UC patients using RS-fMRI, emphasizing the DMN’s role in IBD pathology. Cerebellar disruptions were also observed in studies like Wang et al. (46), who reported functional brain rewiring and decreased cortical stability in UC patients, underscoring the cerebellum’s involvement in neurophysiological changes related to IBD. Goodyear et al.[45]used multimodal MRI analysis to identify deep gray matter changes associated with IBD, further supporting the idea that subcortical structures and cerebellar regions may be affected during disease progression. Some studies also documented limbic hyperconnectivity during active disease. Patients experiencing flares showed increased connectivity between the hippocampus-caudate and amygdala-anterior cingulate pathways. This corticolimbic hyperactivity was linked to anxiety severity and working memory deficits, indicating the limbic system’s involvement in inflammation-related neuropsychiatric symptoms. Nonetheless, some inconsistencies require further investigation. For instance, Thomann et al. (48) examined combined patterns of brain structure and function using multimodal data fusion. Still, the variability in findings regarding the extent and nature of functional disruptions across different studies raises important questions. Specifically, the relationship between these neural changes, disease severity, and clinical symptoms remains unclear. These contradictions emphasize the need for more comprehensive research to understand better the neurobiological mechanisms behind IBD and its associated emotional and cognitive issues. Future studies could help clarify the connection between gut health and brain function, ultimately aiding in the development of improved patient management strategies.

## 7 Discussion

This review investigates changes in resting-state connectivity in patients with UC, a condition that has received limited research attention compared to CD in previous studies. Therefore, this review provides an overview of several studies that have employed RS-fMRI analysis techniques in the context of UC. The studies in the literature have employed diverse methodologies to evaluate resting-state FC using the RS-fMRI BOLD signal in patients with UC.

Few studies have focused primarily on alterations in the plasticity of specific brain regions (33, 45, 46). The cumulative findings from the reviewed studies indicate significant functional alterations in distinct brain regions among individuals with UC. These connectivity pattern alterations are seen in the cerebellar and salience networks, VISN, and DMN of patients with UC when compared to HCs, as shown in the previous section. Few studies (33, 45) reported that patients with IBD have increased FC in and between the limbic and basal ganglia structures compared to HCs. Moreover, patients with active UC have increased FC between the hippocampus and the caudate (33).

Among the limited studies that directly compare UC to CD, those reporting significant differences between the groups indicate that UC is associated with increased resting activity and FC across regions in the occipital, parietal and frontal areas (31, 48). Although Fan et al. (33) restricted their UC participants to those in an active disease state, research on resting state activity in UC has not yet delineated differences between various states of disease activity. Building on robust evidence characterizing the specificity of RS-MRI alterations in UC and CD, additional investigations could evaluate whether these disparities are unique to IBD or demonstrate broader generalizability across immune-mediated inflammatory disorders, such as rheumatoid arthritis.

Disruptions in limbic network connectivity, especially insula hyperconnectivity, along with activity in the anterior cingulate cortex and amygdala, are strongly associated with the severity of anxiety and depression in UC patients as reported using RS-fMRI (46). Several MRI studies on patients with anxiety and depression show structural and functional abnormalities in the limbic system and frontal lobe (49, 50). Anxiety and depression may exacerbate chronic health conditions via different mechanisms, including altering the hypothalamic–pituitary–adrenal axis, increasing sympathetic activity, decreasing vagal tone and suppressing the immune system (16). As two studies reported (33, 45), abnormalities in the limbic system’s anatomical structure and function may contribute to unstable mood states, including an increase in anxiety and depressive disorders, in patients with UC. However, one study has not found a correlation between alterations in the brain regions associated with emotion regulation and UC clinical measurements (47). From these studies’ findings, neural patterns could serve as an early biomarker to guide proactive mental health screening and personalized treatments. For example, patients showing these patterns might benefit from neuromodulation techniques (such as transcranial magnetic stimulation targeting the anterior cingulate cortex or anti-inflammatory biologics), which could help break the brain-gut-inflammatory cycle that worsens mood disorders.

One of the key strengths of using fMRI in UC research is its ability to characterize, in a non-invasive manner, the central mechanisms or neurofunctional changes related to the disorder in patients with clinically confirmed diagnoses. They can detect subtle brain abnormalities that may not be evident on structural MRI or other imaging modalities, offering valuable insights into the neural underpinnings of UC. Another advantage of fMRI techniques is their capacity to capture real-time brain activity, which facilitates the investigation of dynamic brain processes, such as social interaction, attention and language.

It is important to highlight some key limitations of this review. Notably, while disease activity or severity metrics are routinely collected in UC studies, none of the UC-focused investigations operationalized these variables as group contrasts or predictors within their RS-fMRI statistical models. This review does not conduct a systematic review or meta-analysis due to substantial variations in the included studies’ inclusion and exclusion criteria and analytical methods. There are also several limitations related to the methodological design. For example, most of these studies involved relatively small sample sizes, which can affect the generalizability of their findings to broader populations. Additionally, heterogeneity in disease activity is observed in most studies. All studies exclusively included right-handed participants, overlooking neurobiological differences in left-handed individuals.

## 8 Future study directions

Future research should build on the current literature and tackle the previously mentioned limitations. The sample size limitations pose significant challenges, which could be addressed by using multiple databases, data sharing, data augmentation and transfer learning, as well as by including diverse populations. Integrating fMRI with other modalities, such as machine learning and radiomics, could help better understand the neurobiological underpinnings of UC and its comorbidities. Additionally, most of these studies included only right-handed participants, so future studies should compare right- and left-handedness. Future studies examining FC in patients with UC could benefit from using a non-gastrointestinal chronic inflammatory disease patient group as a comparator to elucidate the relationship between RS-FC and gastrointestinal and systemic inflammation. Another notable finding of this review is the lack of longitudinal studies investigating FC or resting-state activity in UC or IBD in general. Adopting longitudinal research designs could provide critical insights into the temporal alterations in brain structure and function in UC. The design of this is a crucial focus for advancing UC research. Serial RS-fMRI assessments across different disease stages from active flare to remission and relapse would enable dynamic neural mapping and help clarify causal relationships between central nervous system changes and gut inflammation. Such research could identify predictive biomarkers for treatment response and disease progression, potentially transforming personalized management strategies.

The present findings may provide valuable insights into the etiological processes contributing to UC pathogenesis and its neuropsychiatric manifestations. Furthermore, it would provide critical insights into the developmental and age-related changes in resting-state brain function in individuals with UC, an area that has not been extensively researched. This review encompasses research involving known subtypes of IBD. Future research should also incorporate investigations that do not specify a particular subtype, such as those conducted by Wang et al. and Deng et al. (42, 51).

Findings from RS-fMRI research may have significant implications for clinical applications in IBD, especially in informing the design of future therapeutic interventions. For instance, identifying brain imaging biomarkers associated with depression in IBD could facilitate the customization of pharmacological therapies or non-invasive brain stimulation techniques, such as transcranial direct current stimulation, for individuals suffering from depression related to IBD. Integrating multidimensional datasets and applying integrative analytical frameworks can be strategically employed to investigate complex associations among neuroimaging modalities (e.g., structural/fMRI), neuropsychological assessments, peripheral biomarkers of inflammation, genomic profiles, and other variables. Such approaches enable a comprehensive exploration of cross-domain interactions, advancing mechanistic insights into the interplay between neurobiological systems, behavioral phenotypes, and molecular pathways. Clarifying pathophysiological disruptions in bidirectional brain-gut signaling pathways in IBD represents a critical scientific priority, independent of evolving research trajectories examining baseline cerebral functional architecture.

A growing body of research indicates that artificial intelligence (AI) and machine learning (ML) methods can be used to analyse images, thereby enhancing diagnostic accuracy for IBD diseases and predicting clinical outcomes (52–55). These techniques also offer valuable predictive insights into postoperative outcomes (56). For example, deep learning models, such as convolutional neural networks (CNNs), are used in endoscopic images to differentiate UC from CD with high accuracy and reduced reading time (57). When integrated with fMRI methods, recent advancements in these techniques have been developed to diagnose autism spectrum disorder and have demonstrated promising results (58). Therefore, these techniques can help address issues arising from RS-fMRI analysis.

## 9 Conclusion

fMRI enhances our understanding of the disease’s underlying neuropathological processes in patients with UC. Its non-invasive nature allows for patient assessment without the need for task performance, making RS-fMRI a valuable methodological approach. The literature investigating differences in resting-state function and connectivity in UC reveals significant variability in methodologies, particularly concerning participant characteristics, variables of interest and analytical approaches. This review identified significant structural and connectivity differences in various brain regions in patients with UC compared to HCs. Further research Building on recent advancements, subsequent studies should prioritize larger, more diverse cohorts to enable robust analyses and integrate multi-dimensional methodologies. Additionally, longitudinal studies are essential to clarify unresolved questions regarding the correlation between RS-fMRI alternation and clinical parameters such as disease activity in UC.
